# TAPAS: Towards Automated Processing and Analysis of multi-dimensional bioimage data

**DOI:** 10.12688/f1000research.26977.1

**Published:** 2020-10-28

**Authors:** Jean-François Gilles, Thomas Boudier

**Affiliations:** 1 Sorbonne Université, Institut de Biologie Paris-Seine, IBPS, Paris, France; 2 Institute of Molecular Biology, Academia Sinica, Taipei, Taiwan

**Keywords:** Image processing, image analysis, automation, OMERO, ImageJ, Fiji

## Abstract

Modern microscopy is based on reproducible quantitative analysis, image data should be batch-processed by a standardized system that can be shared and easily reused by others. Furthermore such system should require none or minimal programming from the users.

We developed TAPAS (Towards an Automated Processing and Analysis System). The goal is to design an easy system for describing and exchanging processing workflows. The protocols are simple text files comprising a linear list of commands used to process and analyse the images. An extensive set of 60 modules is already available, mostly based on the tools proposed in the 3D ImageJ Suite.

We propose a wizard, called TAPAS menu, to help the user design her protocol by listing the available modules and the parameters associated. Most modules will have default parameters values for most common tasks. Once the user has designed her protocol, she can apply the protocol to a set of images, that can be either stored locally or on a OMERO database.

An extensive documentation including the list of modules, various tutorials and link to the source code is available at https://imagej.net/TAPAS.

## Introduction

Modern microscopy, through new systems like light-sheet or high-throughput microscopes, is generating a vast amount of complex data that needs to be analysed. These data can be large in size or in number. Furthermore, for the purpose of reproducible quantitative analysis, these data should be batch-processed by a standardized system, that can be easily shared and reused. Some batch systems already exist such as CellProfiler ^1^, ICY ^2^ protocols or ImageJ macros ^3^. However, these systems may require some programming knowledge or time to set up for inexperienced users or are not yet fully multi-dimensional.

In the last 10–20 years, and more recently with deep-learning methods, a lot has been accomplished in the field of image processing, especially for image segmentation. However, there is no real standardization for image analysis protocols. Arganda-Carreras and Andrey ^4^ designed a first version of a systematic image analysis pipeline. Furthermore, due to the recent advances in fast volumetric microscopy, more and more data are produced, but it lacks a systematic way of organizing raw data and subsequent analysed data and results. With the spread of database systems such as OMERO ^5^, more and more imaging facilities and labs are storing their data in a more organized fashion.

## Methods

We developed TAPAS (Towards an Automated Processing and Analysis System) as a system for describing and exchanging processing workflows. The protocols are simple text files comprising a linear sequence of commands. An extensive set of 60 commands is already available, mostly based on the 3D ImageJ Suite ^6, 7^. The design of the protocol allows simplified tracking of processed data and quantitative results, by using keywords to design the image data, such as ? *image*?.

TAPAS is focusing on data organization rather than complex segmentation or analysis algorithms. TAPAS focused originally on data stored on an OMERO database, by allowing to retrieve, perform classical segmentation procedures and analysis, and push back the results, both images and tables, to the database. Data on OMERO are, by design, organized by user, then *projects* and *datasets*. In TAPAS the current analysed image is simply referred by the keyword *?image?*, and the corresponding project and dataset the data belongs to by *?project?* and *?dataset?* respectively. Subsequent processed data are then referred as *?image?-processing*, for instance *?image?-nucleus* for the result of nucleus segmentation. Similarly, additional datasets can be created such *?dataset?-labels* to store the results of segmentation. The results tables can be stored using the name of the image as reference such as *?image?-nucleus-volume.csv*. Results tables will be linked to the original raw image using OMERO *attachments*.

### Implementation

The system is implemented in Java, with a core library, including OMERO and BioFormats input/output utilities, and a plugins library including a comprehensive set of modules. Each module is generic as it will process a generic *Image* class, and each class will get an *Image* as input and will return an *Image* as output. Parameters are managed as simple *String* files, allowing flexible management of parameters, even for inexperienced Java programmers. The current system uses the ImageJ *ImagePlus* class as implementation for the *Image* class, however any other class can be used, allowing the use of any Java library.

The processing pipeline is constructed as an ordered list of processing classes, with their corresponding parameters. Since the classes are generic, a processor is also specified as how to process the image data, by default an *ImagePlus* processor is built. An experimental version of processor with a set of processing modules using the *clearCLBuffer* class has been tested and validated using the CLIJ system ^8^.

### Operation

**Figure 1.**
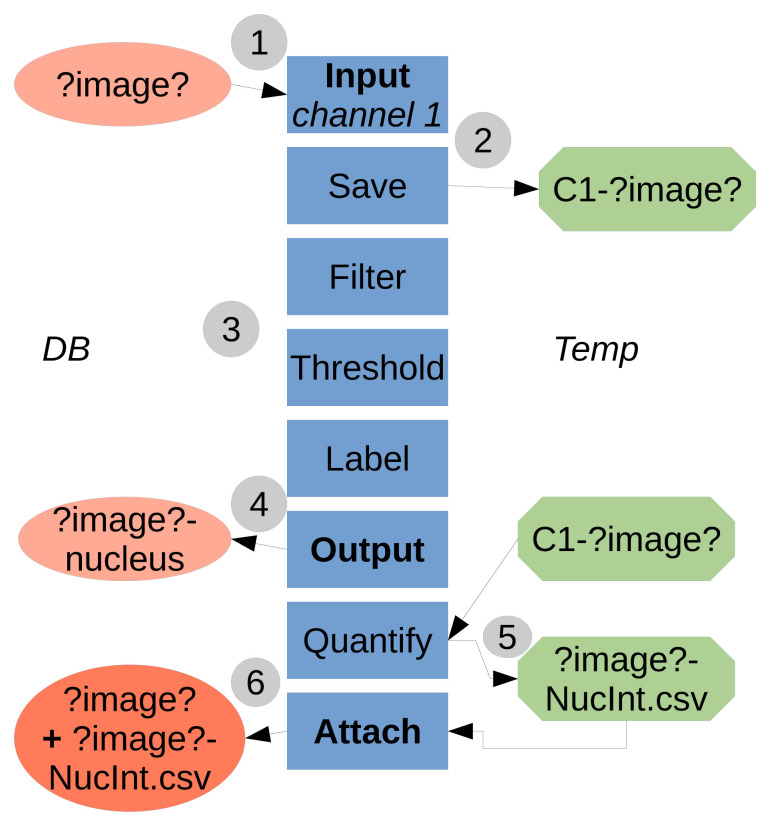
Flowchart of the TAPAS system. **1**) The data to be processed is **input** into the processing pipeline from the Database (either OMERO or local). *?image?* is a keyword used to refer to the image name. The names in boxes refer to the module names. **2**) The necessary data to be used later is saved locally, in a temporary folder (home folder, ImageJ/Fiji folder or system temporary folder). Here we saved the raw data for channel 1. **3**) The data is processed, here a classical pipeline consisting of filtering, thresholding and labelling. **4**) The resulting labelled data is **output** to the Database, here the labelled structure for channel 1 is the nucleus. **5**) The previously saved raw data is used as parameter to quantify intensity inside the labelled nuclei. The results table is saved first in a file locally. **6**) The results table file is then **attached** to the original processed image. The temporary saved data (raw data for channel 1 and results table file) can be then deleted within the pipeline or manually.

TAPAS is java-based and works with the ImageJ/Fiji system, the use of a OMERO database is optional. TAPAS is designed to work with a database; either OMERO or a local database. A local database is a folder organized, not unlike OMERO, as projects, datasets, and finally images. We also add an attachments' folder to store the results tables. Having a completely similar organization between OMERO and a local database allows to have an exact same protocol to run using an OMERO database or a local database. A typical workflow of the system is presented in Figure 1.

## Use case

The system separates the data to be processed from the processing pipeline. Firstly, the list of image data to be processed is built, each image data to be processed is identified by its project, dataset and name (either on OMERO or on a local DB), and by the channel and frame to be processed. Second, the processing pipeline file is to be selected. After clicking *run*, the system will process the images sequentially, displaying information for each module, and the final processing time per image. Raw data will be *pulled* from the database and processed and analysed data will be *pushed* back to the database.

We propose a simple TAPAS *menu* that will display in an organized manner the list of available modules with their corresponding category and documentation. After selecting a module, the list of parameters will be displayed, the user can then manually enter the parameters values, and the corresponding processing pipeline text will be created.

## Conclusion

TAPAS is a comprehensive system for data processing automation, relying on an extensive set of more than 60 modules for processing and analysis of multi-dimensional image data. An extensive documentation including the list of modules, various tutorials and links to the source code is available at https://imagej.net/TAPAS.

## Data availability

All data underlying the results are available as part of the article and no additional source data are required.

## Software availability

Software available from: https://imagej.net/TAPAS.

Source code available from: https://github.com/mcib3d/tapas-core/.

Archived source code at time of publication: http://doi.org/10.5281/zenodo.4091177 ^9^.

License: GPL 3.0
