# TAPAS: Towards Automated Processing and Analysis of multi-dimensional bioimage data

**DOI:** 10.12688/f1000research.26977.2

**Published:** 2021-07-02

**Authors:** Jean-François Gilles, Thomas Boudier

**Affiliations:** 1 Sorbonne Université, Institut de Biologie Paris-Seine, IBPS, Paris, France; 2 Institute of Molecular Biology, Academia Sinica, Taipei, Taiwan

**Keywords:** Image processing, image analysis, automation, OMERO, ImageJ, Fiji

## Abstract

Modern microscopy is based on reproducible quantitative analysis, image data should be batch-processed by a standardized system that can be shared and easily reused by others. Furthermore, such system should require none or minimal programming from the users.

We developed TAPAS (Towards an Automated Processing and Analysis System). The goal is to design an easy system for describing and exchanging processing workflows. The protocols are simple text files comprising a linear list of commands used to process and analyse the images. An extensive set of 60 modules is already available, mostly based on the tools proposed in the 3D ImageJ Suite.

We propose a wizard, called TAPAS menu, to help the user design the protocol by listing the available modules and the parameters associated. Most modules will have default parameters values for most common tasks. Once the user has designed the protocol, he/she can apply the protocol to a set of images, that can be either stored locally or on a OMERO database.

An extensive documentation including the list of modules, various tutorials and link to the source code is available at https://imagej.net/TAPAS.

## Introduction

Modern microscopy, through new systems like light-sheet or high-throughput microscopes, is generating a vast amount of complex data that needs to be analysed. These data can be large in size or in number. Furthermore, for the purpose of reproducible quantitative analysis, these data should be batch-processed by a standardized system, that can be easily shared and reused. Some batch systems already exist such as CellProfiler ^1^, ICY ^2^ protocols or ImageJ macros ^3^. However, these systems may require some programming knowledge or time to set up for inexperienced users or are not yet fully multi-dimensional.

First category of automation methods is script-based automation, such as ImageJ or ICY scripts, both based on Java or JavaScript language. These scripts will include reference to location of data itself and hence are not easily shared between users. Furthermore, inexperienced users may have difficulty writing their own workflow.

Second category is graph-based automation, such as ICY protocols or CellProfiler. Although they are quite easy to apprehend for inexperienced users, it may not be straightforward, due to the intrinsic graph structure of the workflows, to build complex processing pipelines. In case the workflows can be exported as text files, they are usually very complex and not easily readable and modifiable.

Although, a lot has been accomplished in the last 10–20 years, and more recently with deep-learning methods, in the field of image processing, there is still a lack of standardization for image analysis protocols. Arganda-Carreras and Andrey ^4^ designed a first version of a systematic image analysis pipeline. Furthermore, due to the recent advances in fast volumetric microscopy, more and more data are produced, but it lacks a systematic way of organizing raw data and subsequent analysed data and results. With the spread of database systems such as OMERO ^5^, more and more imaging facilities and labs are storing their data in a more organized fashion.

## Methods

We developed TAPAS (Towards an Automated Processing and Analysis System) as a system for describing and exchanging processing workflows. The protocols are simple text files comprising a linear sequence of commands. An extensive set of 60 commands is already available, mostly based on the 3D ImageJ Suite ^6, 7^. 

TAPAS modules are organised by categories:

- Input/Output, to download and upload image and quantitative results data.
- Calibration, to load, save and apply calibration to image data.
- Processing, to edit image data such as cropping, mathematical operations, scaling.
- Filtering, to filter 2D or 3D image data using either 3DFastFilters from the 3D ImageJ Suite or CLIJ filters (GPU filtering).
- Thresholding, to create binary images, using different methods such as automatic global thresholding, hysteresis thresholding, iterative thresholding, percentile thresholding.
- Labelling, with minimal and maximal size filtering.
- Post-processing, to filter out binary or labelled data, including mathematical morphology (fill holes, closing), exclude objects on edges, keep biggest object, filter objects based on size or shape.
- Analysis, including geometrical and shape measurements, intensity measurement, co-localisation, numbering, distances.
  Advanced analysis including layer analysis.
- Measurements processing, such as merge and append results tables.
- Utilities including running a macro or a local command line program.

The design of the protocol allows simplified tracking of processed data and quantitative results, by using keywords to design the image data, such as ?i *mage*?. Although TAPAS includes advanced segmentation and analysis modules, TAPAS is focusing more on data organization and simplified workflows rather than complex segmentation or analysis algorithms. TAPAS focused originally on data stored on an OMERO database, by allowing to retrieve, perform classical segmentation procedures and analysis, and push back the results, both images and tables, to the database. Data on OMERO are, by design, organized by user, then *projects* and *datasets*. In TAPAS the current analysed image is simply referred by the keyword *?image?*, and the corresponding project and dataset the data belongs to by *?project?* and *?dataset?* respectively. Subsequent processed data are then referred as *?image?-processing*, for instance *?image?-nucleus* for the result of nucleus segmentation. Similarly, additional datasets can be created such *?dataset?-labels* to store the results of segmentation. The results tables can be stored using the name of the image as reference such as *?image?-nucleus-volume.csv*. Results tables will be linked to the original raw image using OMERO *attachments*.

The TAPAS workflow language is based on a linear sequence of modules, described by their name and parameters values. The module name is simply preceded by the keyword *process*. Note that the values for parameters can include space characters, and eventually commas if the parameter value is to be a list. A typical example for one module is then:



          // comment for the module
process : name_of_module
name_parameter1 : value of the parameter1
name_parameter2 : value of parameter2
        


The main difference with other workflow systems is that, in TAPAS, there is only one image data that is transferred between the different modules, and this image data does not need to be explicitly defined as one of the parameter. There is hence an implicit link between the output of a module and the input of the next module. Each module will process the current image and return a new image that will become the current image.

The other main idea of the TAPAS workflow is that additional image data that may be needed in the workflow, such as raw data for intensity measurement, must be saved locally first, and then used as a parameter in a module. A typical example will be detailed later.

### Implementation

The system is implemented in Java, with a core library, including OMERO and BioFormats input/output utilities, and a plugins library including a comprehensive set of modules. Each module is generic as it will process a generic *Image* class, and each class will get an *Image* as input and will return an *Image* as output. 

The current system uses the ImageJ *ImagePlus* class as implementation for the *Image* class, however any other class can be used, allowing the use of any Java library. Alongside the actual image data, that is passed from module to module, the information about the original image data is also passed to the module in the form of an *ImageInfo*. This information will store the original information about the storage location of the image, i.e. its original *project*, *dataset* and *image* name, as defined above. The information about the processed *frame* or *channel* is also stored, or set to 1 if no specific channel or frame is defined for the current image. 

Parameters are managed as simple *String* files, allowing flexible management of parameters, even for inexperienced Java programmers.

Parameters are defined by a pair of key-value, the key being the name of the parameter and the value being the actual value of the parameter as a *String* value. Note that, for sake of simplicity and genericity, no types are defined for the parameters, the developer has to take care of the correct values to be entered. 

In order to create a new module, the developer has first to create the processor module that will define the *Image* class that will be used, or use the already provided *ImagePlus* processor. It is as simple as writing:

*public class TapasProcessorMyImageType extends TapasProcessorAbstract<MyImageType>*

Second the developer has to implement the functions of the generic processing class. The function *getName* will simply return the name defined for the module. The main function is *execute* that will effectively do the processing of the image, and will take the *Image* type defined in the processor as input and output. The function *setCurrentImage* will transfer the image information to the module as an *ImageInfo*, and does need any rewriting. This information may be used to input or ouptut data.

Finally, three functions deal with the parameters, the first one *setParameter* will give the information about one parameter to the module in the form of two *Strings*, the name and the value of the parameter. Actually, this function, besides *execute*, may need some work if the developer wants to carefully check the parameter names and their corresponding values. Usually the set of possible parameter names are hard-coded and defined within the module class itself. Two other functions *getParameters* and *getParameter(parameter name)* are used for displaying parameters information. 

In order to link the Java classes to the modules names, there is a simple text file called *tapas.txt* organised as follows: module_name:path.to.the.java.class. The program will check, when initialized, if two classes link to the same name; the program will also store internally in a *hashMap* the link between modules names and java classes. 

The processing pipeline is constructed as an ordered list of processing classes, with their corresponding parameters. Since the classes are generic, a processor is also specified as how to process the image data, by default an *ImagePlus* processor is built. An experimental version of processor with a set of processing modules using the *clearCLBuffer* class has been tested and validated using the CLIJ system ^8^.

### Operation

**Figure 1.**
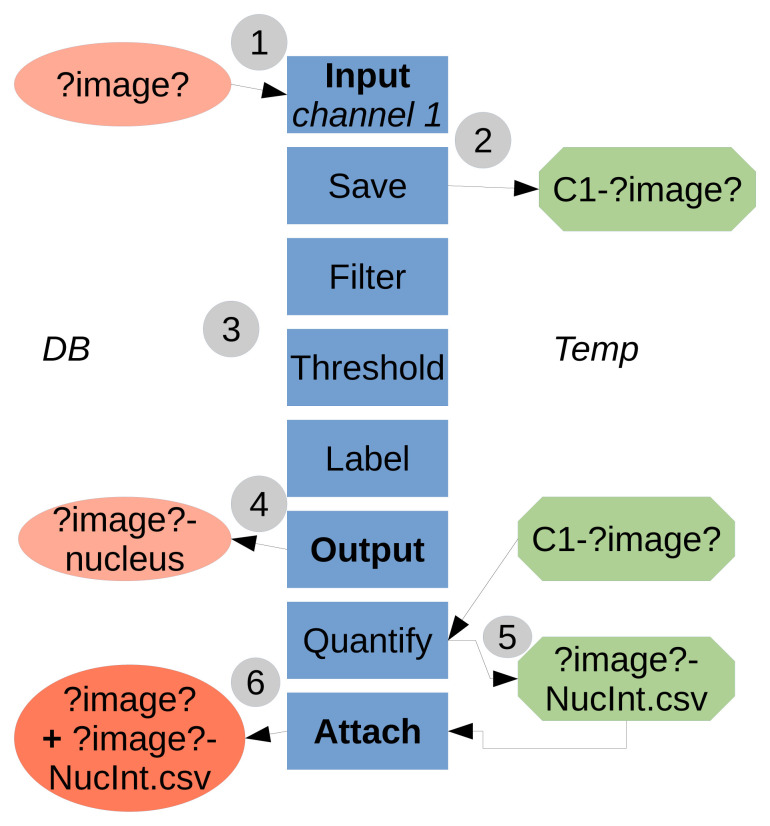
Flowchart of the TAPAS system. **1**) The data to be processed is **input** into the processing pipeline from the Database (either OMERO or local). *?image?* is a keyword used to refer to the image name. The names in boxes refer to the module names. **2**) The necessary data to be used later is saved locally, in a temporary folder (home folder, ImageJ/Fiji folder or system temporary folder). Here we saved the raw data for channel 1. **3**) The data is processed, here a classical pipeline consisting of filtering, thresholding and labelling. **4**) The resulting labelled data is **output** to the Database, here the labelled structure for channel 1 is the nucleus. **5**) The previously saved raw data is used as parameter to quantify intensity inside the labelled nuclei. The results table is saved first in a file locally. **6**) The results table file is then **attached** to the original processed image. The temporary saved data (raw data for channel 1 and results table file) can be then deleted within the pipeline or manually.

TAPAS is java-based and works with the ImageJ/Fiji system, the use of a OMERO database is optional. TAPAS is designed to work with a database; either OMERO or a local database. A local database is a folder organized, not unlike OMERO, as projects, datasets, and finally images. We also add an attachments' folder to store the results tables. Having a completely similar organization between OMERO and a local database allows to have an exact same protocol to run using an OMERO database or a local database. A typical workflow of the system is presented in Figure 1.

TAPAS is using the BioFormats library to open image files so it can handle most common microscopy image files when used with a local database. Common image analysis workflow will require detecting objects in one or several channels, perform analysis in the same or other channels. Most of the time the input data is a multi-channel image, and a specific object type is detected within one channel. However, in TAPAS as in most image processing algorithms, image operations are performed on mono-channel images. The main difference in the TAPAS system is that the workflows are strictly linear and a module can only have one input image. In case a module, such as a co-localisation module, requires two input images, one image will be the main input and the other image will be used as a parameter, and hence need to be saved locally beforehand.

## Use case

To successfully build a TAPAS workflow we propose a simple TAPAS *menu* that will display in an organized manner the list of available modules with their corresponding category and documentation. After selecting a module, the list of parameters will be displayed, the user can then manually enter the parameters values, and the corresponding processing pipeline text will be created. Note that default values are also proposed for most of the parameters, if the user chooses to use the default value for a parameter, the line corresponding to this parameter can be omitted, greatly simplifying the workflow text. 

The system separates the data to be processed from the processing pipeline. Firstly, the list of image data to be processed is built, each image data to be processed is identified by its project, dataset and name (either on OMERO or on a local DB). Second, the processing pipeline file is to be selected. After clicking *run*, the system will process the images sequentially, displaying information for each module, and the final processing time per image. Raw data will be *pulled* from the database and processed and analysed data will be *pushed* back to the database.

We propose a simple TAPAS *menu* that will display in an organized manner the list of available modules with their corresponding category and documentation. After selecting a module, the list of parameters will be displayed, the user can then manually enter the parameters values, and the corresponding processing pipeline text will be created.

Here we describe a typical workflow to perform intensity measurement on detected objects. The first part of the workflow will input the two channels separately, and save the raw intensity of channel 2 locally.



          // input channel 2 and save it locally
// since we are referring to the original image, we only need to specify the channel
process : input
channel : 2
        




          // we save locally in the ImageJ/Fiji folder (other default folders are available)
// ?image? refers to the original image name
process : save
dir : ?ij? 
file : ?image?-C2 
        


Then the channel 1 will be opened, filtered and the nuclei segmented.



          process : input
channel : 1
        




          // 3D median filtering
process : filters
radiusXY : 4
radiusZ : 2
        




          // Otsu global thresholding
process : autoThreshold
method : Otsu
        




          // labelling with a minimal pixel size
process : label
minSize : 100
        




          // and exclude objects touching edges
process : excludeEdges
        


The final labelled nuclei image should be ouptut to the database, in the same dataset and project, a different dataset could be used.

// ?image? refers to the original image name



          process : output
image : ?image?-nuclei
        


Then we can perform geometrical and shape measurement of the nuclei, and *attach* them to the original image.



          // we perform the measurements and save them locally first
process : measurement
list : volume, compactness
dir : ?ij?
file : ?image?-nuclei-measurements.csv
        




          // we attach the results file to the original image
process : attach
dir : ?ij?
file : ?image?-nuclei-measurements.csv
        


We do the same for intensity quantification, using the original raw data of channel 2 as parameter for the signal image. The current image in the workflow is the labelled nuclei image. The quantification results will be attached to the original image.



          // we use the saved raw C2 channel as image for raw signal quantification image, we saved the quantification results locally first. 
process : quantification
dirRaw : ?ij?
fileRaw : ?image?-C2
list : mean, sum
dir : ?ij?
file : ?image?-nuclei-quantification.csv
        




          // attach the results to the original image
process : attach
dir : ?ij?
file : ?image?-nuclei-quantification.csv
        


Finally, we can clean-up the ?ij? folder. 



          Process : deleteList
dir : ?ij?
list :  ?image?-C2, ?image?-nuclei-measurements.csv, ?image?-nuclei-quantification.csv
        


## Conclusion

TAPAS is a comprehensive system for data processing automation, relying on an extensive set of more than 60 modules for processing and analysis of multi-dimensional image data. An extensive documentation including the list of modules, various tutorials and links to the source code is available at https://imagej.net/TAPAS.

## Data availability

All data underlying the results are available as part of the article and no additional source data are required.

## Software availability

Software available from: https://imagej.net/TAPAS.

Source code available from: https://github.com/mcib3d/tapas-core/.

Archived source code at time of publication: http://doi.org/10.5281/zenodo.4091177 ^9^.

License: GPL 3.0
