# Supplementary material for: TAPAS: Towards Automated Processing and Analysis of multi-dimensional bioimage data
Source: F1000Res. 2021 Jul 2;9:1278. Originally published 2020 Oct 28. [Version 2] doi: 10.12688/f1000research.26977.2 (PMC8422341; doi:10.12688/f1000research.26977.2)
Supplement: Peer review history

# Peer review: Reviewer response for version 2

**DOI:** 10.5256/f1000research.57741.r88975

**Published:** 2021-09-06

**Authors:** Lit-Hsin Loo

**Affiliations:** 1 Bioinformatics Institute (BII), Agency for Science, Technology, and Research (A*STAR), Singapore, Singapore

The manuscript has been revised to address most of my previous comments. More detailed examples of use cases have been provided in the main text to help the readers better understand the usage of the system.

Is the work clearly and accurately presented and does it cite the current literature?

Yes

If applicable, is the statistical analysis and its interpretation appropriate?

Not applicable

Are all the source data underlying the results available to ensure full reproducibility?

Yes

Is the study design appropriate and is the work technically sound?

Yes

Are the conclusions drawn adequately supported by the results?

Partly

Are sufficient details of methods and analysis provided to allow replication by others?

Partly

Reviewer Expertise:

computational and system biology, image analysis, high-throughput screening and imaging, machine learning, in vitro cell models

I confirm that I have read this submission and believe that I have an appropriate level of expertise to confirm that it is of an acceptable scientific standard.

# Peer review: Reviewer response for version 2

**DOI:** 10.5256/f1000research.57741.r88976

**Published:** 2021-08-20

**Authors:** Robert Haase

**Affiliations:** 1 Cluster of Excellence "Physics of Life", Technische Universität Dresden, Dresden, Germany

I am happy to read the revised version of the manuscript. It covers all my suggestions from the last round. I appreciate the deeper insights into the technology the authors present and the comparison with other open source tools available aiming at a similar target audience. I congratulate the authors to this excellent manuscript and wish the best for the future of the TAPAS project.

Is the work clearly and accurately presented and does it cite the current literature?

Partly

If applicable, is the statistical analysis and its interpretation appropriate?

Not applicable

Are all the source data underlying the results available to ensure full reproducibility?

No source data required

Is the study design appropriate and is the work technically sound?

Yes

Are the conclusions drawn adequately supported by the results?

Yes

Are sufficient details of methods and analysis provided to allow replication by others?

Partly

Reviewer Expertise:

Bio-Image Analysis, Computer Science

I confirm that I have read this submission and believe that I have an appropriate level of expertise to confirm that it is of an acceptable scientific standard.

# Peer review: Reviewer response for version 1

**DOI:** 10.5256/f1000research.29799.r73800

**Published:** 2020-11-18

**Authors:** Lit-Hsin Loo

**Affiliations:** 1 Bioinformatics Institute (BII), Agency for Science, Technology, and Research (A*STAR), Singapore, Singapore

**Summary:**

 Gilles *et al.* report a new software tool called TAPAS for batch processing and analysis of microscopy images. The tool is based on ImageJ/Fiji and can load images from the local file system or remote OMERO database. A set of 60 analysis modules or commands are currently available, mostly come from a previously developed package, 3D ImageJ Suite.

 The ability to batch process images using a standardized system is critical for ensuring the reproducibility of published results, and also the continuity of the development and applications of established analysis pipelines. The authors correctly point out that there are many existing tools that can do batch processing, such as CellProfiler, Icy, or ImageJ macros. However, one of the key advantages of TAPAS is the simplification of the specifications of an analysis workflow using text files (which can be edited using any text editor) and intuitive command syntax (which require minimum effort to learn). Thus, TAPAS can still fill a gap currently unaddressed by these other tools in the field.

 Overall, the work is technically sound and the article is well written. However, insufficient details and examples or use cases have been given to demonstrate the applications of TAPAS. Also, the targeted applications and the current limitations of the software are not well explained or discussed.

**Major comments:**

1. For this reviewer, the syntax of TAPAS is one of the most interesting and novel parts of the software. However, insufficient details and examples have been provided to explain the syntax and rules of the “TAPAS language”. One suggestion is to provide an example for a typical image analysis pipeline (e.g., image import, preprocessing, segmentation, feature extraction, result export). The authors may include pseudo codes (or a block diagram) and the corresponding TAPAS code that can accomplish the tasks.
2. It is not very clear what types of microscopy images can TAPAS process. The authors kind of alluded that the tools is meant for 3D images, but this has not been clearly presented. For example, can TAPAS process bright-field or RGB images? Time-lapse images? Or 3D-stack images?
3. Can TAPAS run any ImageJ commands/functions? Or is it restricted only to functions from the ~60 modules?
4. The authors may present some simple examples of the results and screenshots that can be obtained from TAPAS. For example, counting of cells or measurement of protein expression levels in 3D images. These examples will be very helpful to let the readers understand how TAPAS may be used.
5. Another major advantage of using text-file based configurations is that the configurations may be version-controlled using standard software such as Git. Perhaps the authors may consider adding this function in the future.

**Minor comments**

1. In the abstract, “Once the user has designed her protocol, she can … “ may be changed to “he/she”.

Is the work clearly and accurately presented and does it cite the current literature?

Yes

If applicable, is the statistical analysis and its interpretation appropriate?

Not applicable

Are all the source data underlying the results available to ensure full reproducibility?

Yes

Is the study design appropriate and is the work technically sound?

Yes

Are the conclusions drawn adequately supported by the results?

Partly

Are sufficient details of methods and analysis provided to allow replication by others?

Partly

Reviewer Expertise:

computational and system biology, image analysis, high-throughput screening and imaging, machine learning, in vitro cell models

I confirm that I have read this submission and believe that I have an appropriate level of expertise to confirm that it is of an acceptable scientific standard, however I have significant reservations, as outlined above.

# Peer review: Reviewer response for version 1

**DOI:** 10.5256/f1000research.29799.r73799

**Published:** 2020-11-02

**Authors:** Robert Haase

**Affiliations:** 1 Cluster of Excellence "Physics of Life", Technische Universität Dresden, Dresden, Germany

The authors present TAPAS which is an approach Towards an Automated Processing and Analysis System. One can see it as a workflow-design tool, primarily, but not exclusively, to the ImageJ 3D Suite. 

 The authors' efforts are appreciated because TAPAS fills a gap in the ImageJ/Fiji ecosystem. Furthermore, TAPAS is extensible and user friendly. TAPAS allows the removal of technical implementation details from the process of designing an image processing workflow. Furthermore, users are suggested to organize their data in a structure similar to OMERO databases. Standardization in image data organization is also a field where every step forward is deeply acknowledged.

 For the presented manuscript, I would suggest minor additions: First of all, a more detailed report of the state-of-the-art would be interesting. Differentiation of TAPAS against Icy Protocols ^1^ and Knime ^2^ could give new insights to the reader. Furthermore, also commercial systems such as Zen and Apeer (Zeiss, Jena, Germany) and Arivis (Arivis, Munich, Germany) come nowadays with workflow design user interfaces which might be worth exploring and distinguishing.

 From the user's perspective, I would love to see an example workflow and an explanation of how the user benefits from exploiting the capabilities of TAPAS in a practical context. The TAPAS website contains examples and tutorials and the manuscript could benefit from this amazing documentation.

 On the implementation side, I would happy to read some insights about how plugins for TAPAS can be implemented and distributed to collaborators. I think if the involved procedures would be explained a bit more in detail, developers could estimate efforts to implement TAPAS wrappers for their tools and algorithms, e.g. for denoising and segmentation. This increases the chance of other developers picking up TAPAS as a distribution system of their tools.

 Furthermore, on the technical side, I wonder if it is possible to implement processing steps that take two images from two former processing steps as input. Figure 1 suggests TAPAS is a linear system where each operation has just one input and one output. I'm not sure if I interpret this correctly.

 The fact that TAPAS is developed fully in the open endorses the authors as fantastic contributors to the bio-image analysis open-source community. The audience knows from former projects like the authors' famous ImageJ 3D Suite that long-term support is available for many years. I think potential future plans for TAPAS could be mentioned in the manuscript. To my experience, communicating long-term perspectives clearly to the community pays off, because if users know that long-term support is given, the chance is higher that they pick up a new tool and build their workflows with it.

 To conclude, TAPAS is a great extension for ImageJ/Fiji and eases integration of workflows in the OMERO ecosystem. It is extensible and flexible in use giving a powerful toolbox in the hands of end-users.

Is the work clearly and accurately presented and does it cite the current literature?

Partly

If applicable, is the statistical analysis and its interpretation appropriate?

Not applicable

Are all the source data underlying the results available to ensure full reproducibility?

No source data required

Is the study design appropriate and is the work technically sound?

Yes

Are the conclusions drawn adequately supported by the results?

Yes

Are sufficient details of methods and analysis provided to allow replication by others?

Partly

Reviewer Expertise:

Bio-Image Analysis, Computer Science

I confirm that I have read this submission and believe that I have an appropriate level of expertise to confirm that it is of an acceptable scientific standard, however I have significant reservations, as outlined above.

## References

[1] : Icy: an open bioimage informatics platform for extended reproducible research.*Nat Methods*.2012;9(7) : 10.1038/nmeth.2075690-610.1038/nmeth.207522743774

[2] : KNIME: The Konstanz Information Miner.2008; 10.1007/978-3-540-78246-9_38319-32610.1007/978-3-540-78246-9_38
